# Therapeutic potential of *Bacopa monnieri* extracts against hepatocellular carcinoma through *in-vitro* and computational studies

**DOI:** 10.1371/journal.pone.0321445

**Published:** 2025-04-28

**Authors:** Awais Altaf, Asia Kiran, Muhammad Sarwar, Tahir Maqbool, Sumaira Sharif, Hana’a Iqbal, Saba Farooq, Qurban Ali, Shiming Han, Ajaz Ahmad

**Affiliations:** 1 Institute of Molecular Biology and Biotechnology, The University of Lahore, Lahore, Pakistan; 2 Faculty of Health Sciences, Equator University of Science and Technology, Masaka, Uganda,; 3 National Institute of Virology, International Center for Chemical and Biological Sciences, University of Karachi, Karachi, Pakistan; 4 Department of Plant Breeding and Genetics, University of the Punjab, Lahore, Pakistan; 5 School of Biological Sciences and Technology, Liupanshui Normal University, Liupanshui, China; 6 Department of Clinical Pharmacy, College of Pharmacy, King Saud University, Riyadh, Saudi Arabia; University of Alabama at Birmingham, UNITED STATES OF AMERICA

## Abstract

**Background:**

Among various cancers, primary liver cancer is the seventh most diagnosed malignancy and is the second most prevalent contributor to cancer-causing deaths. During conventional treatment, the recurrence of disease, low drug inefficacy, and severe side effects are the main limitations. Recently, natural anticancer medicines from the Middle East, Korea, China, Europe, North America, and India have attracted a lot of interest due to their low side effects and better remedial properties. The current study investigated the antioxidative and anticancer effects of ethanolic (BME) and n-hexane (BMH) extracts of *B. monnieri* (L.) Wettst.

**Methods:**

In the current study, phytochemical profiling was done using gas chromatography-mass spectrometry (GC-MS) analysis. The antioxidant potential was measured using DPPH, nitric oxide, superoxide anion, and hydrogen peroxide assays, while the cell viability and apoptotic effect were measured by MTT, crystal violet, and annexin V/PI protocols, respectively.

**Results:**

Higher concentrations of total phenolic contents (274.92±3.52 mgGAE/g), total flavonoid contents (141.99±4.14 mgQE/g) and tannins (55.49±4.63 mgTAE/g) were observed in BME extract with strong antioxidant potential than BMH extract. Also, BME extract showed higher cytotoxicity with less IC_50_ value (24.70 μg/mL) and a lower percentage of cell viability, while the same extract exhibited 58.65% apoptosis against HepG2 cells in comparison to cisplatin and BMH extract. Furthermore, Spiro[(tricyclo[6.2.2.0(2,7)]dodeca-5,9-diene)-4,1’-cyclobutane]-11,2’-dione from BME extract showed the lead docking score of -8.8, -8.1 and -7.8 kcal/mol against TGF-βR1, TNF-α, and iNOS, respectively.

**Conclusion:**

In conclusion, the ethanolic extract of *B. monnieri* has a significant potential for becoming a potent anticancer drug that effectively treats liver damage, including HCC.

## Introduction

Liver cancer is the fourth invasive cancer worldwide, accounting for more than 800,000 mortalities per annum [1]. According to the recent estimates, 830,200 deaths and 905700 cases of liver cancer were observed in 2020. The American Cancer Society estimated that diagnosis of 41,210 cases (27,980 in men and 13,230 in women) and 29,380 fatalities (10,380 cases in females and 19,000 in males) found in 2023 [2]. It is also estimated that 1.4 million expected cases with 1.3 million mortalities will be expected in 2040 [3]. The most prevalent forms of primary liver cancer include hepatocellular carcinoma (HCC), followed by intrahepatic cholangiocarcinoma (10–15%) and other liver malignancies [4]. Almost ninety percent of HCCs have recognized underlying etiologies, including non-alcoholic fatty liver disease, severe alcoholism, and chronic viral hepatitis [5]. Prolonged inflammation, oxidative stress, tissue remodeling, genetic changes, and deregulated cellular signaling are thought to be the important mechanisms involved in HCC development and progression [6]. Recently, the primary treatment options for HCC include surgical resection, chemotherapy, radiotherapy, local ablation therapy, and liver transplantation. These conventional therapies are efficient at early stages of hepatocellular carcinoma but are ineffective at advanced stages due to the development of serious adverse effects such as metastasis, frequent recurrence, drug resistance, and many others [7]. Thus, substantial research is required to discover and develop novel anticancer drugs considering the increasing risk of drug-resistant malignancies with fewer side effects [8]. In the past few decades, considerable attention has been given to medicinal plants and herbal extracts containing valuable phytoconstituents with structural diversity and novel therapeutics to inhibit or destroy cancerous cells by overcoming drug resistance during cancer treatment [9]. According to the World Health Organization (WHO), more than eighty percent of the global population opts for these traditional medicinal plant-based treatment options [10]. Medicinal plants possess a wide range of bioactive compounds such as catechins, polyphenols, flavonoids, triterpenoids, alkaloids, glycosides, and a plethora of others, which may reduce cell proliferation due to their anti-angiogenic, anti-mutagenic, antioxidant and anti-cancer properties [11]. Moreover, plant-derived phytocompounds offer better safety and efficacy because of their distinct molecular characteristics and diverse structure [12].

In medicinal plants, GC-MS analysis is an indispensable technique for identifying therapeutically important compounds [13]. In this context, molecular docking is an economical and efficient method for testing and developing natural medicines. This method provides information on the interaction between natural ligands and receptors, which may be used to predict how the drug will attach to target proteins to elucidate the underlying biochemical processes that are disrupted in pathological conditions [14]. Over many years, the concept of inflammation-induced cancer has been recognized, and numerous research has proven that pro-inflammatory cytokines in tumor microenvironment influence every stage of tumor development and treatment efficacy[15]. These inflammatory cytokines are considered a promising target in several cancer treatments. Among various cytokines, TNF-α and TGF-β are essential in playing different functions at various stages of tumor development [16]. TNF-α is an important inflammation-causing cytokine, mostly produced through macrophages, although it is also secreted by various malignant cells that promote tumor metastasis, migration, proliferation and invasion through activating TNF-α-induced PKCα-, NF-кB, and AP-1signaling pathway [17]. Previous research has shown that serum levels of TNF-α and other pro-inflammatory cytokines were considerably greater in HCC patients compared to healthy individuals; hence, elevated TNF-α levels were linked to HCC progression and development [18]. Notably, TGF-β has a dual function in cancer development. At early stages, it inhibits cell proliferation by initiating apoptosis in cancer cells. While at later stages, it promoted tumor growth by inducing angiogenesis, immune suppression, and epithelial-mesenchymal transition (EMT) in hepatocellular carcinoma by activating the TGF-β/Smad pathway [19]. A high-throughput proteomics analysis of early HCC tissue samples demonstrated that invasive HCC tissues were found to have a significant expression of TGF-β levels [20].

Furthermore, nitric oxide is generated by endothelial NOS (eNOS), neuronal NOS (nNOS) and inducible NOS(iNOS) and is involved in the initiation and development of cancer [21]. Excessive or prolonged NO synthesis has been linked with various characteristics of tumor formation, such as genomic instability, angiogenesis, chemoresistance, and metastasis [22]. Elevated levels of NO (1200 μmol/L) were observed in HCC patients before undergoing radiofrequency thermal ablation than control (22 μmol/L).

*Bacopa monnieri*, locally known as Brahmi, belongs to the Scrophulariaceae family and is a small creeping herb that grows in moist areas throughout Europe, Australia, India, Asia, Africa, and South and North America [23]. *B. monnieri* is an excellent reservoir of bioactive compounds, including bacoside A & B, triterpenoid saponins, nicotine, betulinic acid, stigmasterol, bacopasides I & II, herpestine, D-mannitol, cucurbitacin, and several others are extensively investigated for their antioxidant, nootropic, bactericidal, anti-inflammatory, analgesic and anticancer properties [24]. Extensive research and clinical studies support these conventional pharmacological assertions. Traditionally, it is widely used to treat a variety of oxidative stress-related health disorders, such as neurological disorders, malignancies, diabetes, and heart complications [25]. Different *B. monneiri* extracts and their valuable phytochemicals showed promising anti-tumor effects by suppressing proliferation, stimulating apoptosis, and reducing migration and invasion in several human cancer cell lines, including MDA-MB-231 & MCF-7 (breast cancer), HT-29 (colon cancer), DU-145 (prostate cancer) and Cal33 & FaDu (oral cancer)[26, 27]. In another study, it was observed that bacoside A showed an anti-metatastic effect in Wistar albino rats with DEN-induced HCC by downregulating the matrix metalloproteinases (MMP-2 and MMP-9) and lipid peroxidation along with increased antioxidant enzymes levels [28]. Recently, no study has been available in elucidating the therapeutic potential of *B. monnieri* extracts to evaluate the underlying mechanism of TNF-α, TGF-β, and iNOS having a well-established role in HCC development.

In our study, we determined the antioxidative, antiproliferative, and apoptotic activities of *B. monnieri* extracts using various *in-vitro* methods. This is the first-hand report to pinpoint the active anti-HCC ingredients of *B. monnieri* after targeting TNF-α, TGF-β, and iNOS proteins through molecular docking. This study will provide a solid scientific reason for the use of natural alternatives for the HCC treatment, but further research is required to verify the therapeutics of selected phytoconstituents in clinical and *in-vivo* trials.

## Materials and methods

### Reagents

All analytical grade chemicals and solvents were obtained from different companies. 

### Plant extraction

The whole plant of *B. monnieri* (L.) Wettst was purchased from the local herbal market in Lahore, Pakistan. At Government College University (Lahore, Pakistan), a taxonomist identified and gave a voucher number of GC. Herb.Bot-3781 to the plant. It was submitted to the Government College University Herbarium Bank. The plant extracts were prepared according to the protocol given by Al-Nuri [29]. The leaves were dried under shade and were ground into powder by a laboratory grinding mill. The plant was macerated in ethanol (90%, Sigma-Aldrich, USA) and n-hexane (95%, Sigma-Aldrich, USA) solvents for two weeks. After maceration, a rotary evaporator was used to concentrate the filtered solution at 40 ºC (Heidolph Hei-Vap, Germany). This concentrated mixture was achieved with lyophilizer, and a percentage yield of 11.55% of ethanolic and 7.35% of n-hexane extracts of *B. monnieri* was obtained.

### Quantitative estimation of phytochemicals

The concentration of total flavonoids was measured through a colorimetric aluminum chloride assay given by Phuyal and colleagues [30]. The absorbances were taken at 510 nm. The quantity of TFCs (total flavonoids contents) was estimated as mg equivalents of quercetin/g of dry extract using the standard curve obtained from the known concentrations of standard reagent. The concentration of TPCs (total phenolic contents) was measured by the Folin-Ciocalteu procedure [31]. TPCs were noted as mg GAE/g of dry extract). Tannins concentration was estimated following the Folin-Ciocalteu method [32]. Concentrations were estimated as mgTAE/g of dried weight of plant extracts.

### GC-MS profiling of phytocompounds

For GC-MS analysis, lyophilized samples of *B. monnieri* were sent to the ICCBS center (International Centre for Chemical and Biological Sciences) at Karachi University to identify different phytocompounds [33]. The instrument utilized was a TQQQ Agilent (Agilent Technologies, Santa Clara, USA) with a QP-5000 MS (mass spectrometer; quadruple) and a polydimethylsiloxane-clad capillary GC column. As a mobile phase, 99.99% pure helium was used to flow constantly at an average of 1 mL/min. At 250 ºC, the temperature of the injector was maintained with a 1 L injection volume having a 10:1 split ratio. The temperature in the oven was initially operated for 3 minutes at 50 °C, then increased by 7 °C per minute to 280 °C over 25 minutes before being raised to 300 °C (final temperature). For GC–MS spectrum detection, samples were conducted at 40–600 m/z with a scanning period of 0.2 seconds and 70 electron volts of ionization energy. The phytochemicals were detected by comparing the peak height, peak area %, retention time, and the diverse patterns of the mass spectrum of all phytochemicals found in the sample extracts with the spectral database of already available reference compounds present in the NIST library (National Institute of Standards and Technology).

### Estimation of antioxidant activities

#### DPPH scavenging assay.

The antioxidant ability of plant extracts in terms of scavenging free radicals was evaluated in a procedure given by Braca [34]. Firstly, a fresh DPPH stock solution was made with 0.004% w/v in 95 percent methanol. To make stock solutions, 5 mg of ascorbic acid (standard reagent) and plant extracts were mixed in their mother solvents. Different concentrations (50, 100, 150, 200, and 250 μg/mL) of plant extracts, along with reference reagent (ascorbic acid), were used to prepare by sequential dilutions. Each sample (0.1 mL) was combined with DPPH solution (3 mL) and stored in a pitch-dark area for thirty minutes after thoroughly shaking. A control was also prepared using the same reagents but without plant extracts. The optical density of extracts and ascorbic acid was recorded spectrophotometrically with the wavelength of 517 nm against blank. The percent inhibition of the free radicals was calculated by the following equation:

$\text{Free radicals inhibition }\left(\text{\%}\right)\text{= }\left({\text{A}}_{\text{Control}}-{\text{A}}_{\text{Sample}}\right){\text{/A}}_{\text{Control}}\text{×100}$

#### Nitric oxide scavenging assay.

Nitric oxide is formed by Na_2_[Fe(CN)_5_NO] (sodium nitroprusside), and their scavenging potential was evaluated using the method given by Kamble [35]. In the reaction mixture, 1.0 mL of PBS (phosphate buffer saline; pH 7.4), 0.5 mL of sodium nitroprusside (10 mM), 1.0 mL of phosphate buffer (pH 7.4), and 1.0 mL of each sample at varying concentrations ranging from 50 to 250 µg/mL, was kept for 4 hours at ambient temperature. A control was prepared using the aforementioned reagents but without plant extract. The mixture was undergoing centrifugation for 5 minutes at 3000 rpm. Afterwards, 0.5 mL of Griess reagent was mixed with 0.5 mL of supernatant, and results were measured at 546 nm. Ascorbic acid was used as a reference reagent. The reduction percentage of RNS was estimated through the given formula:

$\text{Scavenging of nitric oxide }\left(\text{\%}\right)\text{= }\left({\text{A}}_{\text{Control}}-{\text{A}}_{\text{Sample}}\right){\text{/A}}_{\text{Control}}\text{×100}$

#### Superoxide scavenging assay.

The antioxidant potential of crude extracts to inhibit superoxide anion radicals was evaluated by a previously described procedure [36]. The reaction mixture contains 1.0 mL of both nitroblue tetrazolium [(1M of NBT in PBS (100 mM; pH 7.4) and NADH solution (1M of NADH in PBS (100 mM; pH 7.4)], 1.0 mL of and 0.1 mL of each plant extract or standard reagent (both have concentrations ranging between 50–250 μg/mL). Additionally, 0.1 mL of phenazine methosulphate solution started the reaction and was kept at 20 °C for five minutes. The readings of each sample were taken at 560 nm compared to blank. A control was made with the same reagents except any plant extracts. The following equation was used to determine the scavenging capacity of plant samples:

$\text{Superoxide anion }\left(\text{\%}\right)\text{= }\left({\text{A}}_{\text{Control}}-{\text{A}}_{\text{Sample}}\right){\text{/A}}_{\text{Control}}\text{× 100}$

#### Hydrogen peroxide scavenging assay.

The potential of plant extracts to scavenge hydrogen peroxide radicals was determined using a previously given method [37]. A fresh solution of hydrogen peroxide (2.0 mM) was prepared with the help of phosphate buffer saline (pH 7.4; 50 mM). Briefly, 0.1 mL of sample (at varying concentrations of 50–250 μg/mL) was added into 0.3 mL of PBS and 0.6 mL of H_2_O_2_ solution and left the mixture for ten minutes at 37 °C. The absorbance of all testing samples and ascorbic acid was measured at 230 nm against blank. The scavenging of hydrogen peroxide was calculated by the following equation:


$$\text{Hydrogen peroxide }\left(\text{\%}\right)\text{= }\left({\text{A}}_{\text{Control}}-{\text{A}}_{\text{Sample}}\right){\text{/A}}_{\text{Control}}\text{×100}$$

### Measurement of cytotoxicity.

#### Cell line propagation.

The human liver cancer cell line (HepG2) was obtained from cell line BioBank present at the IMBB (Institute of Molecular Biology and Biotechnology), The University of Lahore, Pakistan. The HepG2 cell line was cultured in DMEM (Dulbecco’s Minimum Essential Medium) that was supplemented with antibiotic (streptomycin/penicillin) and fetal bovine serum (10% FBS). The cells were cultured in a T-75 cell culture flask and incubated at 37 ºC with 5% CO_2_ and 95% air in an incubator (BioTek, Korea). The media was changed every 3–4 days to ensure proper cell growth. The cells were re-cultured after every four to five days [38].

#### Treatment Groups.

Based on treatments, HepG2 cells were segregated into four distinct groups. As a negative control, cells that received DMEM medium were designated as the untreated cells (UT). The other two groups were treated with different BME and BMH extract doses. As a positive control, cisplatin (a commonly used FDA-approved anticancer drug) was given at varying concentrations to the final (fourth) group for comparison.

#### Cytotoxicity assay.

The cytotoxic activity of plant extracts was estimated via the standard protocol of MTT assay [39]. HepG2 cells (1x10^4^ cells/well) were seeded in a 96-well culture plate. After incubation of 24 hours, cells received increasing concentrations (10, 25, 50 and 100 μg/mL) of the plant extracts and cisplatin (positive control) [40], while untreated cells served as negative control received only media. These cells were incubated in a 5% CO_2_ incubator at 37 °C for the next twenty-four hours. After incubation, cells were rinsed using 200 µL of PBS following the removal of media. Then, MTT reagent (25 μL) was introduced in each well and left at 37 °C for 3 hours. Then, the formazan crystals were dissolved in DMSO after removing MTT dye. The results were taken at 570 nm using a microplate spectrophotommeter (BioRad, Singapore). Every experiment was conducted in triplicates. The non-linear regression analysis was utilized to calculate the IC_50_ values using a graph pad prism (version 5.03).

#### Morphological examination.

To observe the therapeutic impact of BME and BMH extracts on the cancer cell morphologies, 1x10^4^ cells were cultivated in a 96-well flat bottom plate. The cultured cells were exposed to different doses (10 μg/mL, 25 μg/mL, 50 μg/mL and 100 μg/mL) of both plant extracts and cisplatin for 24 hrs, and the images of morphological changes were observed using a microscope [35].

#### Determination of viable cells using crystal violet assay.

The percentages of adherent cells, or live HepG2 cells, were measured by crystal violet staining [41]. After the cultivated cells were seeded in a 96-well plate, they were treated for 24 hours with various IC_50_ concentrations of crude extracts and cisplatin, which were calculated using the MTT assay. Following treatment, the medium was removed using PBS solution, and 0.05 mL of crystal violet dye (0.5%) was introduced in each well for staining live cells. These plates were left at room temperature for 10 minutes. Then, the excess stain was washed out with PBS. After drying overnight, the extra stain was removed from the treated cells by applying 50 µL of ethanoic acid (10%). The absorbances were estimated at 600 nm. The percent HepG2 cell viability was estimated after the administration of plant extracts and cisplatin in the following way:


% of live cells = Treated cells/ Untreated cells x 100

#### Evaluation of apoptosis using annexin V/PI Assay.

To estimate the apoptosis effect induced by the extracts in cancer cells, the apoptotic activity was measured following the kit manufacturer’s instructions (annexin V/PI) (Merck-Millipore; MCH100105) [42]. HepG2 cells (5x10^5^ cells per well) were grown on 12-well plates and then exposed to respective IC_50_ concentrations of cisplatin and extracts for treatment. The cancer cells were centrifuged for 5 mins at 1000 rpm, washed with PBS, and put in 1x binding buffer (100 µL). Then, propidium iodide (10 µL) and annexin V-FITC binding (5 µL) were used to stain the suspended cells and left at room temperature for 15 minutes. The automated cell Muse Analyzer (Merck-Millipore) was used to measure the outcomes of cell death induction. 

### Computational study

#### Selection of hepatotoxic target proteins.

Three-dimensional TNF-α, TGF-βR1 and iNOS structures, having the PDB IDs 2AZ5 (only chain A), 1RW8 (only chain A), and 4NOS, respectively, were retrieved from Protein Data Bank. These proteins were selected as a molecular target for this investigation because these are implicated in many processes of liver disorders that, if not treated at an early stage, may develop into hepatocellular carcinoma. Targeting these proteins may reduce the severity of various advanced hepatic complications.

#### Proteins preparation.

The selected target proteins were prepared by eliminating undesired co-crystallized ligands and water molecules before the addition of partial charges allocated to atoms, Gasteiger charges, Kollman charges and polar hydrogen using Discovery Studio [43]. The prediction of active sites is to determine the precise functional region of the protein to comprehend its activity. The active amino acid residues in binding pockets of target receptors were inferred using the literature survey or using PYMOL [44]. Specified dimensions of different grid boxes for target macromolecules are depicted in Table 1.

**Table 1:** The dimensions of the grid box for different selected target proteins are given as follows.

Target proteins	Center			size			Exhaustiveness
	X	Y	Z	X	Y	Z	
TNF-α(Tumor necrosis factor-alpha	-18.486	72.735	38.929	40	40	40	8
TGF-βR1 (Transforming growth factor-beta receptor 1)	7.327	17.295	17.025	40	40	40	8
iNOS (inducible nitric oxide)	4.035	95.635	20.795	40	40	40	8

#### Preparation of ligands.

Different phytocompounds were screened in both extracts of *B. monnieri* using GC-MS analysis to investigate the potent hepatoprotective drug candidates. The structures of identified compounds were downloaded in SDF files from the PubChem or ChemSpider databases and saved in a pdb file. The ligands were optimized through energy minimization and changed to a Pdbqt file by utilizing the AutoDock Vina program for further analysis [45].

#### Selection criteria for bioactive phytocompounds.

The drug-likeness of identified phytochemicals was predicted using SwissADME software. The drug-likeness of a phytoconstituent depends on following Lipinski’s rules. SwissADME (https://www.SwissADME.ch) is a tool to investigate different parameters of Lipinski’s rules [46]. PkCSM (https://biosig.lab.uq.edu.au/pkcsm/prediction) and admetSAR (http://lmmd.ecust.edu.cn/) are free online software that was used for various toxicity parameters assessment. Hepatotoxicity was assessed using the pkCSM program [47], whereas acute oral toxicity, carcinogenicity mutagenicity, and acute oral toxicity variables were determined utilizing the admetSAR tool [48]. Compounds were selected based on drug-like properties for molecular docking to identify a safe drug. To predict these parameters, the software was uploaded with the canonical SMILES of the ligands. This computational study excluded the natural chemicals that had one or more violations of Lipinski’s rules or displayed a positive sign for any toxicity parameter. The filtered compounds were further subjected to molecular docking against selected target proteins.

#### Molecular docking.

The drug-like ligands and proteins were prepared using the AutoDock vina tool (version 1.5.7), which reduced energy and set a grid box to cover all amino acids in the active site defined by co-crystallization. Both molecules (proteins and ligands) were converted and saved in a pdbqt format [49]. Vina used a global optimization algorithm to accomplish docking, taking information from both input files (proteins and ligands) and specified dimensions listed in the configuration file of the grid box. After connecting with the target receptors, nine different conformations were generated for each ligand; the best binding affinities were used to select the ideal position [50]. Macromolecules were taken as rigid entities during the docking process, and different conformations were produced by maintaining the flexibility of ligands. The lowest favorable binding energy establishes the stable association between the target receptor and ligand, having an RMSD value <1Å (Angstrom), which is defined as its lowest favorable binding energy. The lowest binding affinities and the molecular interactions of phytocompounds with active site residues of target proteins were analyzed in detail compared to standard drug [51]. To form a stable docked complex, the target macromolecule was aligned with a stable conformer that was selected based on best binding affinities [52]. PYMOL (version 2.5.4) was utilized to analyze the post-dock outcomes by further examining and visualizing the docked complexes. The 2-dimensional and 3-dimensional images were captured by BIOVIA Discovery Studio (client 2021) [53].

#### Analysis of interactions.

The ligand-protein interactions are involved in forming stable docked complexes and are measured using a web server protein-ligand profiler [54].

#### Prediction of pharmacokinetic properties.

Usually, the selected drug candidates were subjected to the ADMET analysis (absorption, distribution, metabolism, elimination, and toxicity) using different online software to predict their safety in animal and human systems. The initial toxicological study of the expected candidate is a fundamental step in designing and developing a safe and effective medicine before undergoing a pharmacological trial [55]. We used the PkCSM tool for early-stage toxicological and pharmacokinetic profiling of selected plant-based chemicals [56].

#### Statistical analysis.

All the data was evaluated by one-way ANOVA (analysis of variances) with post-hoc Tukey’s test for comparison between treatment groups. For data analysis and IC_50_ calculations, GraphPad Prism (version 5.03) and Statistix (version 8.0) software were used for data analysis. Results were given as mean ± Standard deviation of three independent experiments (n=3). It is observed that analysis was statistically significant at *p-value* < 0.05. Before performing ANOVA, homogeneity of variance and normal distribution of data were evaluated using Bartlett’s and Shapiro-Wilks tests.

### Ethical statement

It has been confirmed that the experimental data collection complied with relevant institutional, national, and international guidelines and legislation with appropriate permissions from authorities of the Institute of Molecular Biology and Biotechnology, The University of Lahore, Lahore 54300, Pakistan.

## Results

### Quantitative phytochemical estimation

The concentration of important pharmacologically active agents in each plant extract was also measured quantitatively (Table 2). For all crude extracts, TFCs (total flavonoid contents) were calculated utilizing the linear regression equation taken from the standard curve of quercetin (y = 0.0023 x - 0.1754, R^2^ = 0.9904). The values of tannins and TPCs (total phenolic contents) in the experimental samples were determined by the tannic acid calibration curve equation (y = 0.0012x + 0.0644, R^2^ = 0.9855) and the standard curve equation of gallic acid (y = 0.0026x + 0.5142, R^2^ = 0.9976), respectively. In general, BME extract exhibited significantly higher contents of flavonoids, tannins, and phenols than BMH extract and has made an important contribution to their biological activities.

**Table 2:** Quantitative evaluation of the phytochemicals screening of both crude extracts.

Sr. no.	Phytochemical Assays	Mean ± SD (n=3)	
		BME	BMH
**1.**	TPC (mgGAE/g DE)	274.92±3.52	154.01±5.38
**2.**	TFC (mgQE/g DE)	141.99±4.14	35.76±1.96
**3.**	Tannins (mgTAE/g DE)	55.49±4.63	22.16±3.33

TPC: Total phenolic contents; TFC: Total flavonoid contents; BME: *B. monnieri* ethanolic extract; BMH: *B. monnieri* n-hexane extract; GAE: gallic acid equivalents; TAE: Tannic acid equivalents; QE: Quercetin equivalents; DE: dry extract. Data was given as the mean±SD of experiments done in triplicates.

### GC-MS investigation

**Fig 1:**
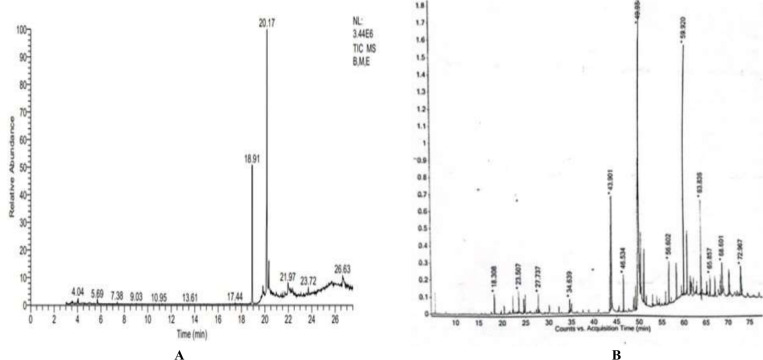
GCMS chromatograms of *B. monnieri* extracts. Different solvents have varying polarities that are depicted with different peak locations and peak lengths in the chromatogram. The solubility of compounds in different solvents also affects their elution times. BME extract (A) identified a diverse array of polar compounds with higher peak lengths and earlier peak locations than BMH extract (B).

GC-MS analysis detected different types and concentrations of natural compounds in both extracts of *B. monnieri*. These identified compounds are given in Fig 1. The secondary metabolites present in both extracts of *B. monnieri* belong to important classes of therapeutically important secondary metabolites such as aromatic hydrocarbons, aliphatic hydrocarbons, fatty acid esters, cholesterol, steroids, terpenoids, phytosterol, vitamins, sesquiterpene and phenolic compounds (Table 3). Most of these compounds were considered medicinally active due to their anti-inflammatory, antioxidative, and anti-tumor attributes.

**Table 3:** GC-MS profiling of both extracts of *B. monnieri.*

GC-MS characterization of ethanol extract of *B. monnieri*						
Peaks No.	Compound’s Name	M.F^A^	M.W^B^	RT^C^	%Area peak	Class of compounds
1.	1,2,3-trimethylbenzene	C_9_H_12_	120.19	3.55	0.83	Aromatic hydrocarbon
2.	Decane	C_10_H_22_	142.28	4.04	1.11	Alkane
3.	Oxiraneoctanoic acid, 3-octyl-, cis-	C_18_H_34_O_3_	298.5	4.45	0.41	Fatty acid
4.	Spiro[(tricyclo[6.2.2.0(2,7)]dodeca-5,9- diene)-4,1’-cyclobutane]-11,2’-dione, 1,3,3,5,12,12-hexamethyl	C_21_H_28_O_2_	312.4	5.02	0.37	Steroid
5.	Tetradecane	C_14_H_30_	198.39	5.69	0.49	Straight chain alkane
6.	Nonadecane	C_19_H_40_	268.5	7.40	0.20	Straight chain alkane
7.	Cholestan-3-one, cyclic-1,2-ethanediyl acetal, (5á)-	C_29_H_50_O_2_	430.7	9.03	0.09	Cholesterol
8.	Tetradecanoic acid, ethyl ester	C_16_H_32_O_2_	256.42	16.97	0.12	Fatty acid ester
9.	Phytol	C_20_H_40_O	296.5	17.46	0.35	Diterpene alcohol
10.	Hexadecanoic acid, 2-methyl-, methyl ester	C_18_H_36_O_2_	284.5	18.91	10.29	Fatty acid ester
11.	9,12,15-Octadecatrienoic acid, ethyl ester, (Z,Z,Z)-	C_20_H_34_O_2_	306.5	20.15	48.61	Fatty acid ethyl ester
12.	2-Monoolein	C_21_H_40_O_4_	356.5	20.99	0.40	Glyceride
13.	[1,1’-Bicyclopropyl]-2-octanoic acid, 2’-hexyl-, methyl ester	C_21_H_38_O_2_	322.5	21.35	0.08	Fatty acid methyl ester
14.	Glyceryl Linolenate	C_21_H_36_O_4_	352.5	22.01	9.95	Fatty acid
15.	Ethyl Linoleate	C_20_H_36_O_2_	308.5	23.07	0.30	Fatty acid ethyl ester
16.	(3E,12Z)-1,3,12-Nonadecatriene-5, 14-diol	C_19_H_34_O_2_	294.5	23.70	1.07	Fatty alcohol
17.	Ethanol, 2-(9-octadecenyloxy)-, (Z)-	C_20_H_40_O_2_	312.5	24.08	0.26	Ether sulphates
18.	Monolinolenin TMS	C_27_H_52_O_4_Si_2_	496.9	24.57	0.35	Fatty acid ester
19.	1-Monolinoleoylglycerol trimethylsilyl ether	C_27_H_54_O_4_Si_2_	498.9	25.69	16.00	Fatty acid ester
20.	Trilinolein	C_57_H_98_O_6_	879.4	26.67	8.73	Triglyceride
**GC-MS characterization of n-hexane extract of** ***B. monnieri***						
**Peaks** **No.**	**Compound’s Name**	**M.F** ^A^	**M.W** ^B^	**RT** ^C^	**%Area peak**	**Class of compounds**
1.	Lupeol	C_30_H_50_O	426.7	72.96	27.83	Triterpene
2.	γ-sitosterol	C_29_H_50_O	414.7	70.20	18.71	Phytosterol
3.	Stigmasterol	C_29_H_50_O	412.7	68.60	23.55	Phytosterol
4.	1-Monolinoleoyl glycerol trimethylsilyl ether	C_27_H_54_O_4_Si_2_	498.9	66.98	6.46	Fatty acid propyl ester
5.	3-Ethyl-5-(2-ethylbutyl)octadecane	C_26_H_54_	366.7	65.85	5.85	Alkane
6.	Vitamin E	C_26_H_54_	366	65.10	5.32	Tocopherol
7.	Hentriacontane	C_31_H_64_	436.8	63.83	26.47	Alkane
8.	Heptacosane	C_27_H_56_	380.7	60.67	11.27	Alkane
9.	Squalene	C_30_H_50_	410.7	59.92	43.42	Triterpene
10.	Diisooctyl phthalate	C_24_H_38_O_4_	390.6	56.60	6.01	Phthalate ester
11.	Ethyl Linolenate	C_20_H_34_O_2_	306.5	20.15	48.61	Fatty acid ethyl ester
12.	2.Monoolein	C_21_H_40_O_4_	356.5	20.99	0.40	Glyceride
13.	Methyl 8-[(1R,2R)-2-[(1S,2R)-2-hexylcyclopropyl]cyclopropyl]octanoate	C_21_H_38_O_2_	322.5	21.35	0.08	Fatty acid methyl ester
14.	Glyceryl liolenate	C_21_H_36_O_4_	352.5	22.01	9.95	Fatty acid
15.	Linoleic acid ethyl ester	C_20_H_36_O_2_	308.5	23.07	0.30	Fatty acid ethyl ester
16.	E,E,Z-1,3,12-Nonadecatriene-5,14-diol	C_19_H_34_O_2_	294.5	23.70	1.07	Fatty alcohol
17.	Emulphor	C_20_H_40_O_2_	312.5	24.08	0.26	Ether sulphates
18.	Monolinolenin	C_27_H_52_O_4_Si_2_	496.9	24.57	0.35	Fatty acid ester
19.	Phytol	C_20_H_40_O	296	43.90	100	Acyclic diterpenoid
20.	Caryophyllene oxide	C_15_H_24_O	220	24.88	2.47	Sesquiterpenes
21.	Toluene	C_7_H_8_	92	5.76	2.17	Organic compound
22.	1-Monolinoleoylglycerol trimethylsilyl ether	C_27_H_54_O_4_Si_2_	498.9	25.69	16.00	Fatty acid ester
23.	Trilinolein	C_57_H_98_O_6_	879.4	26.67	8.73	Triglyceride
24.	Carvacrol	C_10_H_14_O	150	18.30	2.06	Phenol
25.	Lauric acid	C_12_H_24_O_2_	200	23.50	3.74	Fatty acid
26.	Isopropyl palmitate	C_19_H_38_O_2_	298	46.53	9.89	Fatty acid ester

A: Molecular Formula; B: Molecular Weight; C: Retention time (minutes)

*In-vitro* antioxidant potential

#### DPPH scavenging assay.

**Fig 2:**
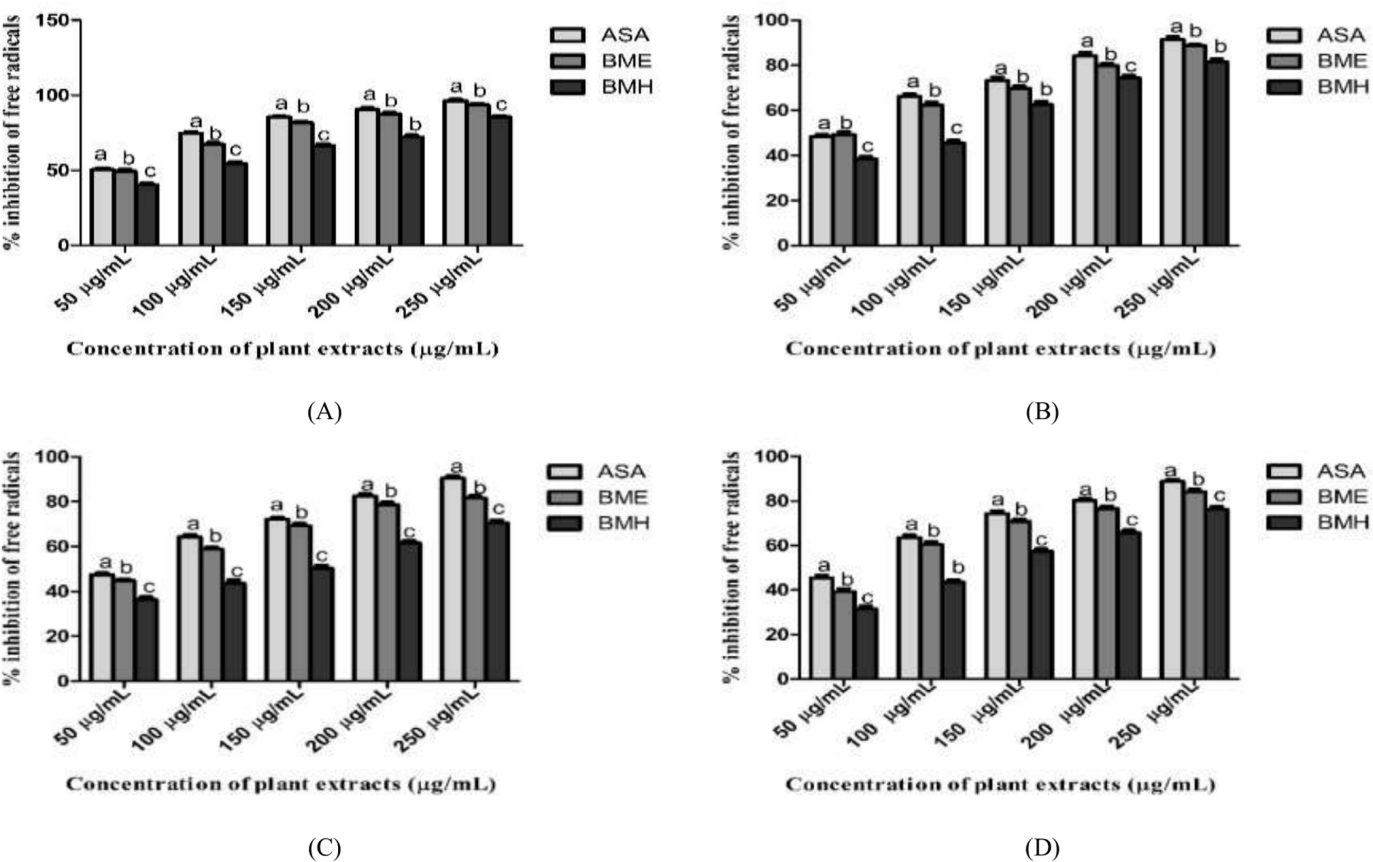
Antioxidant activities of *B. monnieri* extracts (A) DPPH assay (B) Nitric oxide assay (C) Superoxide anion assay (D) H_2_O_2_ assay. One-way ANOVA following Tukey’s test was used to analyze the data and found statistically significant at *p-value* < 0.05. The same letters indicated that the difference was not statistically significant, while different lower-case letters demonstrated statistically significant differences across groups. All experiments were conducted in triplicate, and expressed as mean±SD.

Percentage free radical inhibition for the plant samples and standard antioxidants was calculated using DPPH antioxidant assay. The DPPH scavenging potential of *B. monnieri* extracts was observed to be statistically significant (*p*-value < 0.05) in a concentration-reliant manner (Fig 2A). In Table 4, BME extract showed the best DPPH radical scavenging activity with minimum IC_50_ concentration (26.84 μg/mL) and a higher percentage of free radicals inhibitions, while the weaker scavenging percentage was noticed in BMH extract with the higher IC_50_ concentration of 85.63 μg/mL. Ascorbic acid showed maximum inhibition of free radicals (IC_50_: 13.28 μg/mL) compared to plant extracts. The antiradical potential of plant extracts was found to decrease in the following order: ASA>BME>BMH.

**Table 4:** The antioxidant capacity of both extracts and ascorbic acid.

Sr. no.	Groups	IC_50_ values in μg/mL (n=3) (Mean±SD)			
		DPPH	Nitric oxide	Superoxide anion	Hydrogen Peroxide
1.	ASA	13.28	41.16	47.33	50.83
2.	BME	26.84	46.04	60.95	78.08
3.	BMH	85.63	104.86	135.44	128.21

**ASA**: ascorbic acid (positive standard); **BME:** *B. monnieri* ethanolic extract; **BMH:** *B. monnieri* hexane extract.

#### Nitric oxide scavenging assay.

Each plant extract significantly (*p*-value < 0.05) increased the neutralization of nitric oxide radical with increasing concentrations, as given in Fig 2B. Among both extracts, the BME extract exhibited better scavenging of nitric oxide with an IC_50_ concentration of 46.04 μg/mL in comparison to BMH extract (IC_50_ value: 104.86 μg/mL). The higher inhibiting percentage of oxidant was observed in the case of ascorbic acid (IC_50_ value: 41.16 μg/mL). The decreasing antioxidant potential with increasing IC_50_ values was observed in the following order: ASA<BME<BMH (Table 4).

### Superoxide scavenging assay

Both *B. monnieri* extracts showed scavenging of superoxide anions in a concentration-dependent manner (50, 100, 150, 200 and 250 μg/mL) as depicted in Fig 2C. The antioxidant activity of *B. monnieri* extracts was observed to decrease in the following order: ASA>BME>BMH (Table 4; Fig 2C). BME extract showed stronger superoxide anion scavenging activity (IC_50_: 60.95 μg/mL) than BMH extract with an IC_50_ concentration of 135.44 μg/mL. These extracts showed modest antioxidant activity (*p-value*<0.05) when compared to ascorbic acid (IC_50_: 47.33 μg/mL). Despite this, both extracts act as strong superoxide anion scavengers to neutralize oxidative stress.

### Hydrogen peroxide scavenging assay

The antioxidant potential of plant extracts and standard reagent was found to be higher with increasing concentrations (Fig 2D). Higher percentages of free radical scavenging were observed in BME extract (IC_50_: 78.08 μg/mL) compared to BMH extract (IC_50_: 128.21 μg/mL). Ascorbic acid served as a standard and showed higher antioxidant potential (*p*-value < 0.05) with an IC_50_ value of 50.83 μg/mL compared to both extracts. The decreasing percentage of quenching H_2_O_2_ radicals with increasing IC_50_ values was observed in the following order: ASA>BME>BMH.

### *In vitro* cytotoxic study

#### Determination of cytotoxicity by MTT assay.

**Fig 3:**
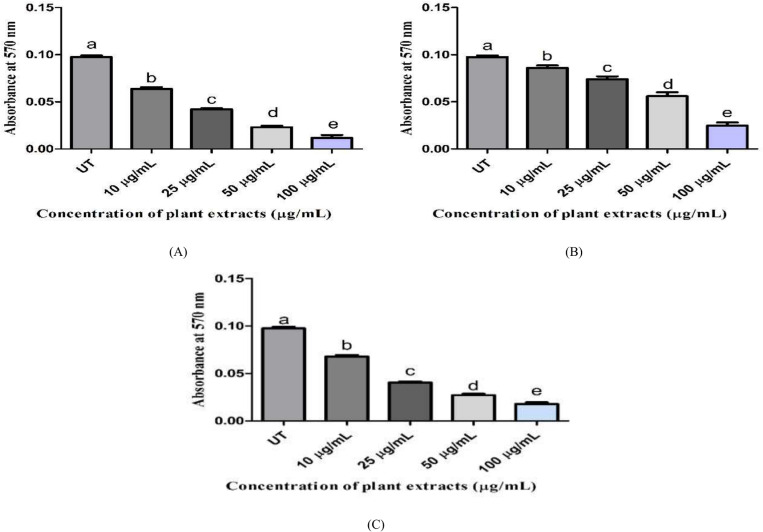
The cytotoxic potential of HepG2 cells treated with plant extracts and cisplatin. (A) HepG2-cells treated with BME extract (B) HepG2-cells treated with BMH extract (C) HepG2-cells treated with cisplatin. Data from triplicate experiments was expressed as mean±SD. One-way ANOVA analysis with Tukey’s tests for multiple comparisons (*p-value* < 0.05) was performed and significant differences between treatment groups were represented by different lowercase letters. UT: untreated cells.

The cytotoxicity of plant extracts in terms of IC_50_ concentrations was evaluated using an MTT assay against the HepG2 cells. The experiment was done with various concentrations of each extract and cisplatin, which served as the reference drug for comparison. The findings showed that increasing the concentration of both extracts and cisplatin causes higher cytotoxicity by reducing the number of viable cells compared to untreated cells (Fig 3). With an IC_50_ value of 24.70 µg/mL, BME extract was shown to be more cytotoxic than BMH extract (41.47 μg/mL), as it significantly reduced the proliferation of malignant cells. Furthermore, cisplatin proved less cytotoxic (IC_50_ value: 25.83 μg/mL) toward cancer cells as compared to BME extract but more active than BMH extract. The IC_50_ values (fifty percent inhibition of cancer cells) of all samples are presented in Table 5. The cytotoxicity of testing samples was increased in HepG2 cells with decreasing IC_50_ values in the following order: BMH>Cisplatin>BME.

**Table 5:** The IC_50_ values of *B. monnieri* extracts against the HepG2 cell line.

Sr. no.	Experimental Groups	IC_50_ concentration in μg/mL
		Cancer cell line (HepG2)
**1.**	BME	24.70
**2.**	BMH	41.47
**3.**	Cisplatin	25.83

IC_50_: inhibitory concentration killing of 50% of the cell population.

#### Morphological investigation.

**Fig 4:**
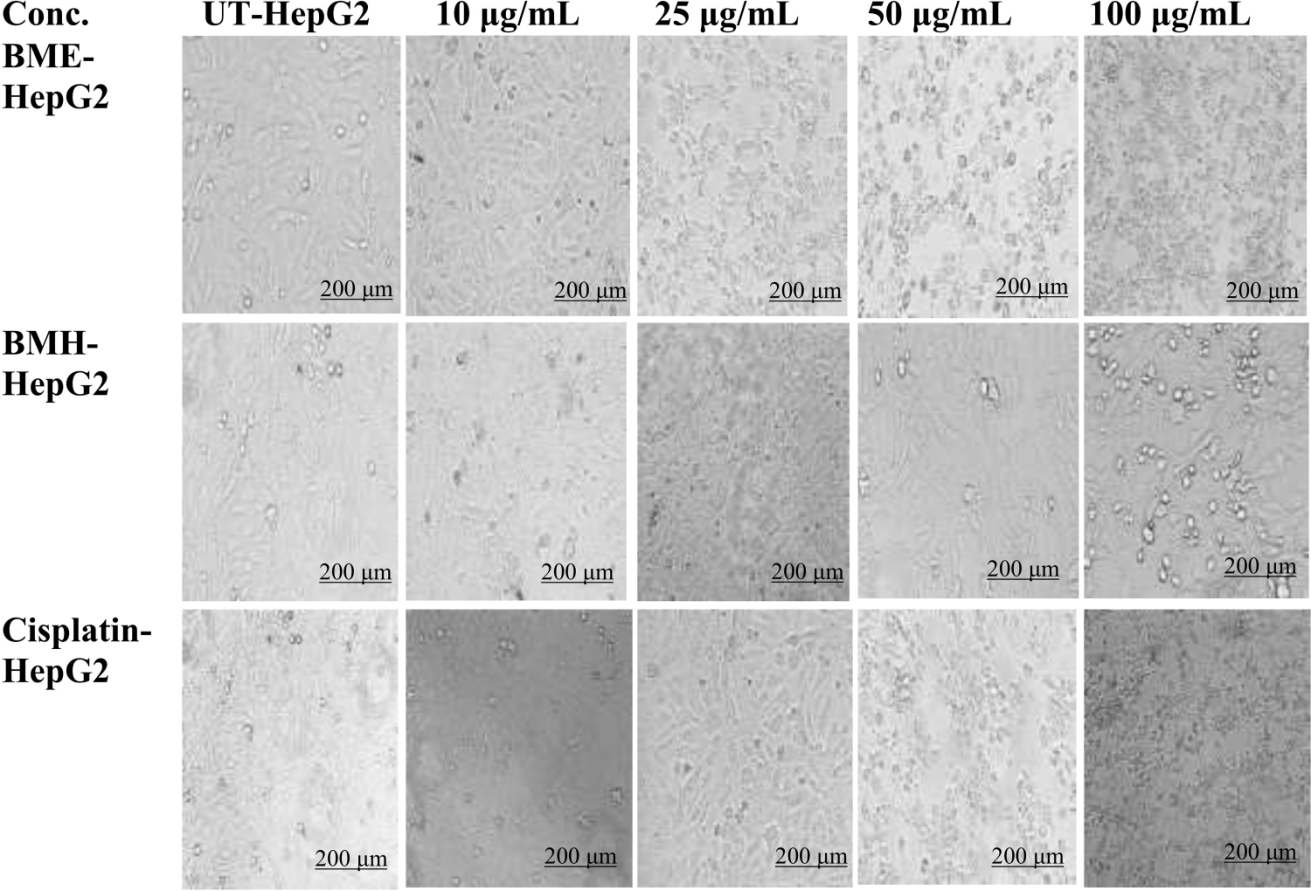
Morphological examination of HepG2 cells on administration of plant extracts and cisplatin. At doses of 25, 50, and 100 µg/mL, BME and cisplatin-induced remarkable morphological changes in cancer cells, whereas BMH extract started the induction of notable morphological changes at 50 µg/mL. BME: ethanolic extract of *B. monnieri*; BMH: *B. monnieri* n- hexane extract of *B. monnieri*; UT: untreated cells. The images were taken at 50× magnifying power using a Floid cell imaging station.

Alterations in the morphology of HepG2 cells following administration of extracts of *B. monnieri* are depicted in Fig 4. Neoplastic cells were observed to change their shape, structure, and size in a dose-reliant manner. At doses of 10, 25, 50, and 100 µg/mL, BME and cisplatin sharply reduced the viability of HepG2 cells along with prominent degenerated cell morphologies. While on administration of BMH extract, any prominent alterations in the shape of viable cells were observed at higher concentrations (50 and 100 µg/mL) when compared to untreated cells.

#### Estimation of cell viability by crystal violet assay.

**Fig 5:**
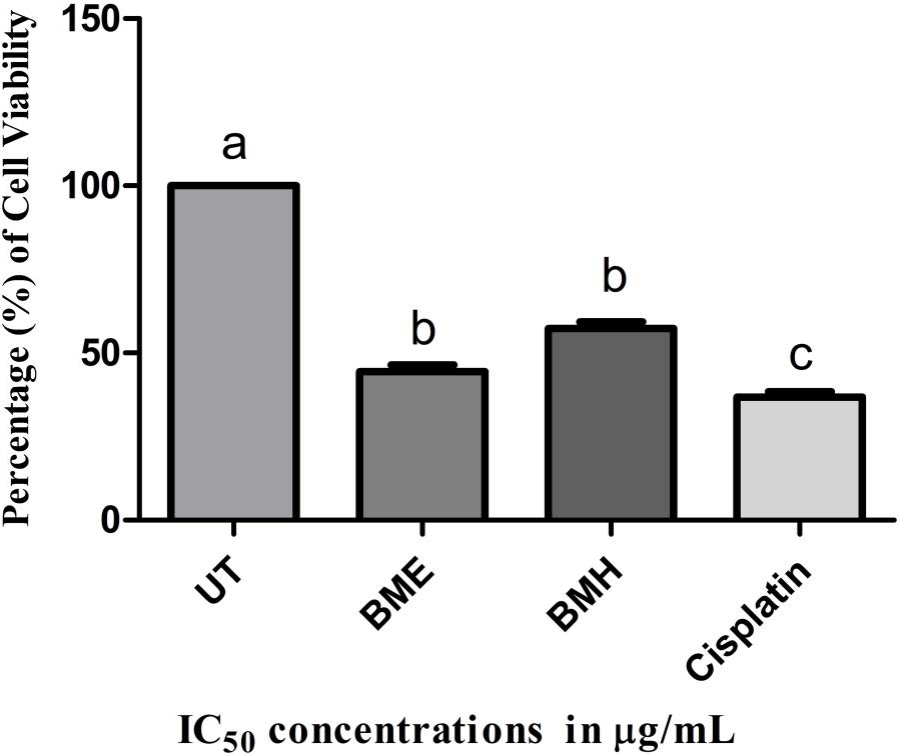
Assessment of the percentage of viable cells via the crystal violet assay. Data from experiments were given as mean±SD (n=3) and considered statistically significant at a *p-value* less than 0.05. Different alphabets showed a significant difference between the treatment groups, and these results were obtained by performing one-way ANOVA with Tukey’s post-hoc test. UT: untreated; BME: *B. monnieri* ethanol extract-treated HepG2 cells; BMH: *B. monnieri* n-hexane extract-treated HepG2 cells; Cisplatin served as a positive control.

Crystal violet assay was used to determine the percentage cell viability of HepG2 cells. Following the treatment with varying IC_50_ concentrations of cisplatin and *B. monnieri* extracts, HepG2 cells were shown to have a higher level of apoptotic activity with the lower percentages of live cells in comparison to the untreated cells. A very small percentage of dead cells were seen in untreated HepG2 cells compared to treated groups (Fig 5). In short, BME extract was found to have a higher inhibitory potential on the multiplication of HepG2 cells with a lower viability percentage compared to BMH extract and cisplatin.

#### Muse analysis via Annexin V/PI.

**Fig 6:**
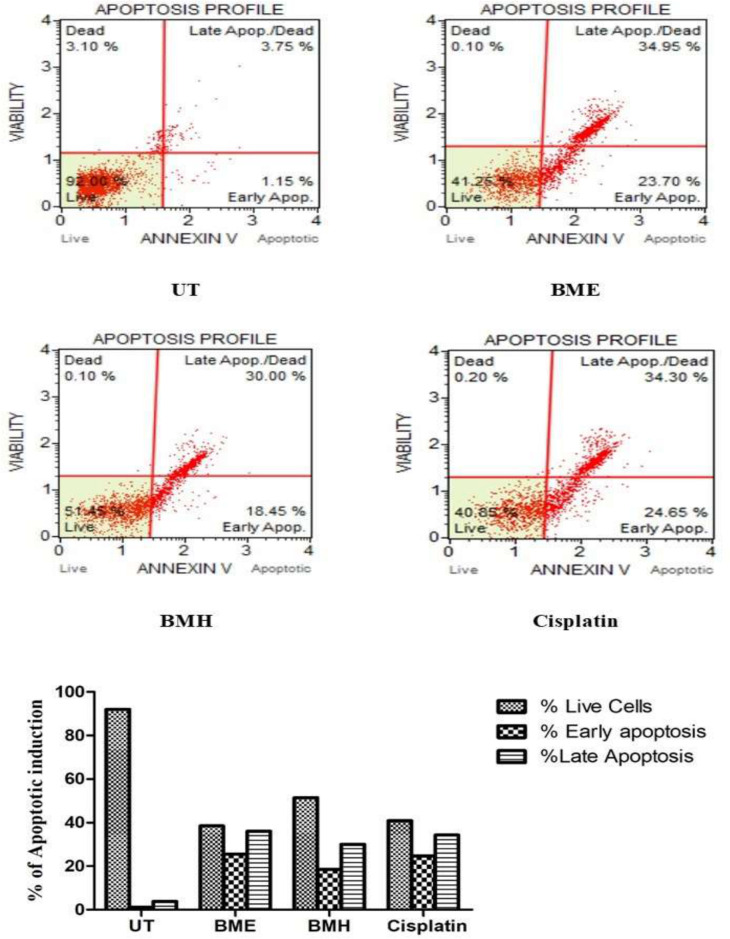
The apoptotic activity of both plants extracts against HepG2 cells. BME: *B. monnieri* ethanol extract; BMH: *B. monnieri* hexane extract; UT: untreated cells.

Annexin V-FITC/PI kit was used to evaluate the apoptotic ability of plant extracts on HepG2 cells. Following treatment with extracts and cisplatin, the percentage of apoptotic induction in HepG2 cells is depicted in Fig 6. In BME-treated HepG2 cells, the higher percentage of apoptosis was 58.65% compared to its BMH extract (48.45%). This may be due to the presence of the higher concentrations of phytochemicals with anticancer potential. The apoptotic percentage of HepG2 cells treated with cisplatin was shown to be 58.95%.

In comparison to untreated cells, all the treatment groups mostly showed an excessive amount of dead cells in their late apoptotic profiles. Following the incubating period, 1.15% of early apoptosis and 3.75% of late apoptosis were observed.

### Computational analysis

#### Selected drug-like compounds.

The phytoconstituents showed at least one violation of Lipinski’s guidelines and a positive result for any of the selected toxicological parameters was removed from the *in-silico* analysis.

Overall, only eight phytocompounds from both *B*. *monnier*i extracts were filtered following Lipinski’s rules and, without exhibiting any kind of toxicity, are listed in Table 6. Molecular docking was performed to assess the anticancer potential of these selected phytocompounds.

**Table 6:** Drug-like compounds selected from medicinal plants following Lipinski’s parameters with satisfying toxicity criteria are given below.

Bioactive Compounds	MW^a^	HBA^b^	HBD^c^	LogP^d^	M.R^e^	L.V^f^	Mutagenicity	Carcinogenicity	Hepato-toxicity	Acute oral toxicity
Epoxyoleic acid	298.46	3	1	3.93	89.38	Yes, 0 Vio	None	None	No	III
Spiro[(tricyclo[6.2.2.0(2,7)]dodeca-5,9- diene)-4,1’-cyclobutane]-11,2’-dione, 1,3,3,5,12,12-hexamethyl (compound A)	312.45	2	0	2.98	93.04	Yes, 0 Vio	None	None	No	III
2-Monoolein	356.54	4	2	4.65	106.20	Yes, 0 Vio	None	None	No	III
Glyceryl linolenate	352.51	4	2	4.81	105.25	Yes, 0 Vio	None	None	No	IV
E,E,Z-1,3,12-Nonadecatriene-5,14-diol	294.47	2	2	4.50	94.35	Yes, 0 Vio	None	None	No	III
Caryophyllene oxide (Compound B)	220.35	1	0	3.15	68.27	Yes, 0 Vio	None	None	No	III
Lauric acid	200.32	2	1	2.70	61.57	Yes, 0 Vio	None	None	No	IV
Toluene	92.14	0	0	1.85	31.41	Yes, 0 Vio	None	None	No	III

**a:** Molecular weight≤500**; b**: Hydrogen bond acceptor≤10**; c:** Hydrogen bond donor≤5**; d:** LogP≤5**; e:** Molar refractivity (40–130)**; f:** Lipinski’s violations.

#### Molecular docking analysis.

**Fig 7:**
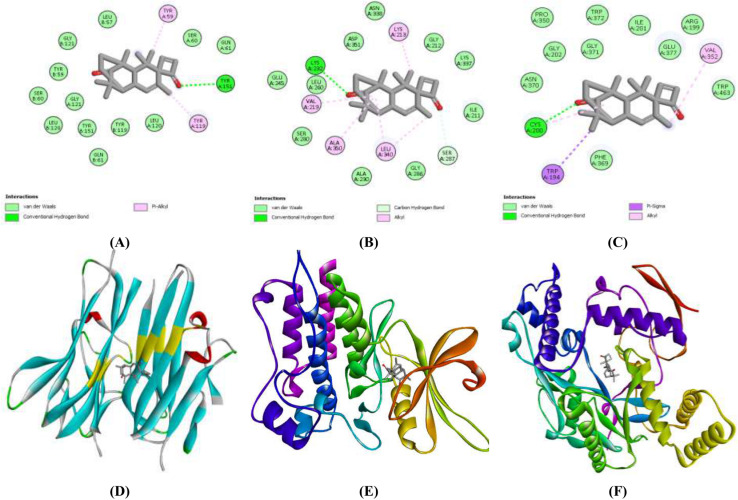
2-D and 3-D images of docked complexes of Spiro[(tricyclo[6.2.2.0(2,7)]dodeca-5,9- diene)-4,1’-cyclobutane]-11,2’-dione against TNF-α (A & D), TGF-βR1 (B &E) and iNOS (C & F).

**Fig 8:**
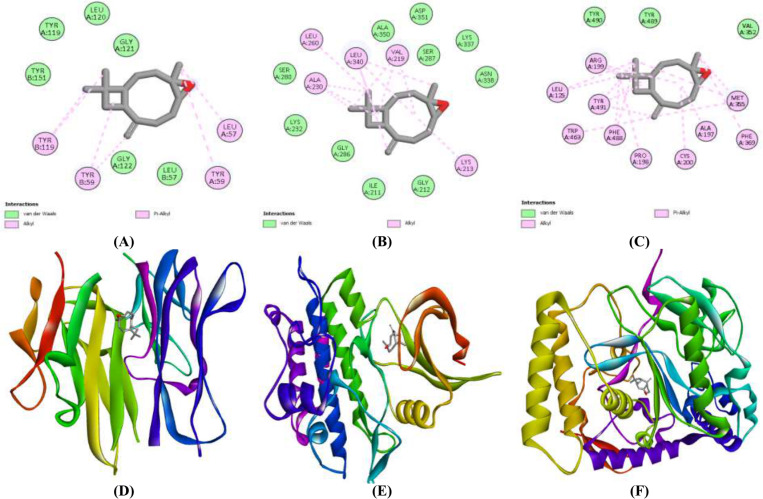
2-D and 3-D images of docked complexes of Caryophyllene oxide against TNF-α (A & D), TGF-βR1 (B & E) and iNOS (C & F).

Among selected compounds of *B. monnieri*, Spiro[(tricyclo[6.2.2.0(2,7)]dodeca-5,9- diene)-4,1’-cyclobutane]-11,2’-dione, 1,3,3,5,12,12-hexamethyl (compound A) screened from ethanol extract, followed by caryophyllene oxide (compound B) taken from n-hexane extract exhibited best docking score against anticancer targets as compared to the sorafenib. Selected drug-like compounds with binding affinities (kcal/mol) are shown in Table 7. Among the best-hits of *B. monnieri*, compound A showed the best binding affinities of −8.1, −8.8, and −7.8 against TGF-βR1, TNF-α, and iNOS as compared to sorafenib. Similarly, compound B exhibited binding affinities of −7.0 kcal/mol for TNF-α, −7.2 kcal/mol for TGF-βR1, and −7.8 kcal/mol for iNOS. This means that both extracts have competent lead compounds for the effective treatment of HCC. The 2-D and 3-D images of top-scorer docked complexes and their molecular interactions are given in **Figs. 7 and 8**. Overall, the BME extract and its active phytochemicals (compound A) with a multi-targeted approach exhibited higher cytotoxic and antioxidative potential by targeting cancer-causing proteins (TNF-α, TGF-βR1 and iNOS) having established role in initiation and progression of HCC. This may be due to the presence of higher concentrations of total flavonoids, phenols and tannins as observed in BME extract than in BMH extract.

**Table 7:** Binding affinities of drug-like phytoconstituents selected from both extracts of *B. monnieri.*

Sr. no.	Selected phytocompounds	TNF-α	TGF-βR1	iNOS
1.	Glyceryl linolenate	−5.6	−6.3	−6.2
2.	2-Monoolein	−5.5	−6.5	−6.1
3.	E,E,Z-1,3,12-Nonadecatriene-5,14-diol	−5.8	−6.7	−6.9
4.	Epoxyoleic acid	−5.3	−6.7	−6.3
5.	Lauric acid	−4.4	−6.0	−5.7
6.	**Spiro[(tricyclo[6.2.2.0(2,7)]dodeca-5,9- diene)-4,1’-cyclobutane]-11,2’-dione, 1,3,3,5,12,12-hexamethyl (Compound A)**	**−8.8**	**−8.1**	**−7.8**
7.	Toluene	−4.5	−5.2	−6.5
8.	**Caryophyllene oxide (Compound B)**	**−7.0**	**−7.2**	**−7.0**
**Standard anticancer drug**	**Sorafenib (anticancer agent)**	**−7.7**	**−7.9**	**−8.4**

#### Molecular interactions of best-hit compounds from *B. monnieri* extracts.

After molecular docking, the lead compounds with high binding affinities with target proteins were selected. The interacting amino acids and their bond lengths implicated in the formation of hydrophobic and hydrogen bonds of top-scorer phytochemicals from both extracts are shown in Table 8, **Figs 7 and 8**. In the flexible environment of protein, hydrogen and hydrophobic interactions are important to establish a stable binding of energetically favorable phytocompounds, which is an important step in identifying the drug lead with improved efficacy. **Compound A** from the BME extract and **compound B** from the BMH extract were found to have higher binding affinities towards TNF-α, TGF-βR1, and iNOS. The conformer A formed hydrogen bonds involving the amino acids, including Tyr 151 in TNF-α, Lys 232 in TGF-βR1, and Cys200 in iNOS.

**Table 8:** Stable molecular interactions of top-scorer compounds with active site residues of target proteins.

Phytocompounds	TNF-α		TGF-β R1		iNOS	
	Interacting residues	Bond length	Interacting residues	Bond length	Interacting residues	Bond length
**Compound A**	**TyrA151,** TyrA 59, TyrB 59, TyrA 119, TyrB 119,	**2.51,** 3.81, 3.73, 3.62, 3.66,	**LysA 232,** LysA 213, ValA 219, LysA 232, LeuA 260, LeuA 340, LeuA 340, AlaA 350, AspA351,	**2.59,** 3.81, 2.95, 3.22, 3.24, 3.36, 3.53, 3.09, 3.55	**CysA 200,** PheA 369, TrpA 463,	**2.69,** 3.46, 3.76,
**Compound B**	LeuB 57, TyrA 59, TyrB 59, TyrB 119,	3.94. 3.33, 3.51, 3.47,	LysA 213, ValA 219, LeuA 260, LeuA 340,	3.73, 3.44, 3.30, 3.58,	LeuA 125, AlaA 197, ArgA 199, PheA 369, PheA 488, TyrA 489, TyrA 491,	3.80, 3.02, 2.96, 3.56, 3.80, 3.89, 3.88,

**Highlighted amino acids**: active residues in hydrogen bonds; **Other amino acids**: active residues in hydrophobic bonds.

Furthermore, the same conformer established hydrophobic interactions with different amino acids: Tyr59, Tyr199 of TNF-α, Lys213, Val219, Lys232, Leu340, Ala350, and Asp351 of TGF-βR1 while Phe369 and Trp463 of iNOS. Moreover, **compound B** only formed hydrophobic interactions with selected target proteins to show their bioactivity. While this phytocompound established several hydrophobic interactions with amino acids of respective target proteins, such as Leu57, Tyr59, and Tyr119 residues of TNF-α, Lys213, Val219, Leu260 and Leu340 of TGF-βR1, and Leu125, Ala197, Arg199, Phe369, Phe488, Tyr 489 and Tyr491of iNOS. Overall, **compound A** of the BME extract exhibited a strong bioactivity with high docking scores compared to other extract. It has been found that lead compounds from BME extract may have the potential to be used in anti-HCC drug development.

#### ADMET analysis of drug candidate.

The pharmacokinetic prediction of the best-hit natural chemicals, including compound A from BME extract and compound B from BMH extract, is depicted in Table 9 as computed by pKCSM. According to a review of the relevant literature, the Ames test is crucial for a compound of interest, and its positive result indicates its mutagenicity and is unable to be further processed as a drug. Both compounds were non-mutagenic. The better aqueous solubility (-4.958), absorption in the human intestine (98.074), and Caco-2 permeability (1.52) were shown by **compound A** than **compound B**. Higher gastrointestinal absorption was shown by each compound, which is important for oral drug absorption into the human body. It was also anticipated that both drug-like compounds would be able to cross the blood-brain barrier and aid in the management of neurological conditions. Both compounds metabolize CYP2D6, CYP3A4, and CYP1A2 during drug metabolism. Similarly, CYP2C19 and CYP2C9 were inhibited by both phytocompounds. No substance was predicted to be an inhibitor of herG-I and herG-II, and it was not involved in cardiotoxicity and hepatotoxicity. Only **compound A** is positive for the OCT2-substrate and readily secreted by the kidney. Both phytochemicals had varying total clearance values, with **compound A** predicted with the value of 0.905 and efficiently eliminated from the body as compared to other compounds (0.445). The maximum tolerated dose observed in humans is -0.488 in **compound A** and 0.148 in **compound B**. This means that **compound B** is well-tolerated in humans before inducing any side effects than compound 1. Moreover, **compound B** exhibited slightly more oral acute rat toxicity with 1.548, and oral chronic rat toxicity with 1.224 as compared to **compound A**. **Compound A** proved slightly more toxic than **compound B** with low values of 0.743 and 0.574 for *T. pyriformis* and minnow toxicity. Overall, to enhance the biological activity and stability of phytochemicals as a potential drug, slight modifications in their native structures are necessary.

**Table 9:** Pharmacokinetic (ADMET) investigation of top-scorer phytochemicals using the PkCSM tool.

Sr. no.	ADMET Variables	Compound A	Compound B
1.	Solubility in water	−4.958	−4.321
2.	Caco-permeability	1.52	1.414
3.	Human intestinal absorption	98.074	91.414
4.	Skin permeation	−3.107	−3.061
5.	P-glycoprotein Substrate	No	No
6.	Blood-brain barrier (BBB)	Yes	Yes
7.	Gastrointestinal absorption (GI)	High	High
8.	Inhibitors of CYP1A2	No	No
9.	Inhibitors of CYP2C19	Yes	Yes
10.	Inhibitors of CYP2C9	Yes	Yes
11.	Inhibitor of CYP2D6	No	No
12.	Inhibitors of CYP3A4	No	No
13.	Total Clearance of a drug	0.905	0.445
14.	Renal OCT2-substrate	Yes	No
15.	AMES test	No	No
16.	Hepato-toxicity	No	No
17.	Maximum human-tolerated dose	−0.488	0.148
18.	Oral acute rat toxicity (LD_50_)	1.695	1.548
19.	Oral chronic rat toxicity	1.643	1.224
20.	Skin sensitivity	No	Yes
21.	Toxicity of *T.Pyriformis*	0.743	1.079
22.	Minnow toxicity	0.574	0.955
23.	Inhibitor of hERG-I	No	No
24.	Inhibitor of hERG-II	No	No

## Discussion

Due to several contributing variables, including genetics and epigenetics, as well as a dearth of effective treatments, liver cancer has emerged as among the main reasons for cancer mortalities globally [57]. Chemotherapeutic drugs are efficacious for hepatocellular carcinoma (HCC) treatment, but their high toxicity, significant side effects, and long-term drug resistance limit clinical outcomes along with increasing death rates [58]. The resurgence in popularity and dependence on medicinal plants as alternative medicines aims to overcome the deficiencies linked with the use of synthetic chemotherapeutic drugs [59]. Various research evidence was reported to bolster the use of traditional medicinal plants in effective HCC regimens [60]. Natural phytochemicals present in medicinal plant extracts activate antioxidant, antitumor, and anti-inflammatory effects, suppressing the signaling pathways responsible for cancer development and promoting mechanisms linked to disease prevention [61]. The interaction of different natural agents with the efficacy of anticancer treatment is well-recognized. Therefore, the current study focuses on alternate natural therapies for the prevention and management of hepatocellular carcinoma. In recent years, medicinal plants with a wide range of bioactive compounds have become popular in the treatment of several inflammation-induced malignancies.

In the present study, BME extract exhibited a higher concentration of TPCs (274.92±3.52 mgGAE/g), TFCs (141.99±4.14 mgQAE/g), and tannins (55.49±4.63 mgTAE/g) as compared to BMH extract. Our results are greater than those of the previously published study on methanolic extract; this discrepancy may be due to changes in plant extract preparation or sample collection methods [62]. The literature revealed that *B. monnieri* methanolic extract showed a higher concentration of these phytochemicals during phytochemical profiling [23]. Phenolic compounds like polyphenols have many medicinal actions, such as being anti-inflammatory, anti-allergic, antibacterial, hepatoprotective, antiproliferative, and cardioprotective. Flavonoids, tannins, terpenoids, and other active metabolites are abundant in plants and have therapeutic properties. These plant-derived active metabolites reduce free radicals and enhance metal chelation [63]. It also protects from severe damage, such as lipid membrane peroxidation, protein deterioration, and DNA mutation brought on by reactive oxygen species [64]. In total, forty-six compounds were screened by GCMS analysis from both extracts of *B. monnieri*. The identified molecules that have been found are part of the significant class of phytochemicals, which includes triglycerides, fatty acid esters, triterpene, steroids, phytosterol, and straight-chain alcohol, among many others. The major components were hexadecanoic acid,2-methyl-, methyl ester, ethyl linolenate, and trilinolein in BME extract, while in BMH extract was squalene, stigmasterol, 1-monolinoleoyl glycerol trimethylsilyl ether, and many others. Hexadecanoic acid,2-methyl-, was reported to have anticancer activity against the HT-29 cancer cell line [65]. Stigmasterol showed an inhibitory effect by causing cell cycle arrest (G1) in endometrial cancer via suppressing IGF1R/mTOR/Akt pathway [66]. A diverse range of bioactive compounds with anti-tumor properties are also present in both extracts. Our results showed that BME extract exhibited a higher level of concentration-dependent antioxidant activity by neutralizing free radicals, including DPPH, NO, H_2_O_2,_ and superoxide anions, than BMH extract, as shown in Fig 2 and Table 4. This may be due to the polarity of ethanol to extract more polar antioxidant phytochemicals, which give better results as compared to other solvents [67]. Similarly, a previous study showed a higher antioxidant activity of methanolic *B. monnieri* extract using various *in-vitro* antioxidant assays [68]. The increased antioxidant activity could be the cause of the higher concentration of bioactive secondary metabolites in their respective extracts.

The therapeutic potential of pharmacologically significant phytocompounds from BME and BMH extracts requires extensive research. Hence, a previous study linked natural agents and conventional plant-based medicines that are useful in treating liver cancer; however, their investigation was restricted to clinical trials [69]. These natural components employ their anti-tumor effect by activating several anti-proliferative processes but usually induce apoptosis in cancerous cells to halt their growth [70]. In the current study, the cytotoxicity of BME and BMH extracts was concentration-dependent, as observed against HepG2 cells using MTT assay (Fig 3). Different inhibitory responses were observed at varying concentrations (10, 25, 50, and 100 μg/mL) of cisplatin, BME, and BMH extracts. The IC_50_ values in terms of cytotoxicity are given in Table 5. According to the American Cancer Research Center, a crude extract is considered a potent cytotoxic agent if its IC_50_ concentration is less than 30 µg/mL [71]. Our results demonstrated that BME extract (IC_50_: 24.77 µg/mL) was observed to be more pronounced than other extracts. These results agree with other studies in which ethanolic and dichloromethane fractions of *B. monnieri* showed good cytotoxicity against breast cancer cell lines [72]. Contrary to our research, *B. monnieri* hexane extract exhibited a better anti-proliferative effect with an IC_50_ value of 53.0 μg/mL on MCF-7 cells [73]. Our findings demonstrated that a higher percentage of apoptosis was induced by BME extract (58.65%) and cisplatin (58.95%) on HepG2 cells in comparison to BMH extract (48.45%) as depicted in Fig 6. Cisplatin can interfere with the DNA repair mechanism by cross-linking purine bases, leading to DNA damage that ultimately activates apoptotic signaling pathways in cancer cells [74]. These findings agree with the preceding research [75]. This is the first report to show the apoptotic induction of *B. monnieri* extracts on HepG2 cells.

Moreover, the cell viability assessment of both extracts was done by crystal violet assay, and the results are presented in Fig 5. The reduced cell proliferation of HepG2 cells was observed at 36.83%, 44.48%, and 57.32% for BME, BMH, and cisplatin, respectively. In both extracts, BME extract demonstrated noticeable cytotoxicity with lower percentage of viable cells. In 2019, the combined effect of bacopaside I and II, isolated from *B. monnieri,* effectively decreased the proliferation, invasion, and migration of MDA-MB-231 & BT-474 through cell cycle arrest and apoptosis [76]. Thus, it is suggested that a significant number of HepG2 cells undergo apoptotic cell death following treatment with BME extract, and this extract may have the ability to develop into a potent anti-cancer drug.

Furthermore, the literature demonstrates that both oxidative stress and inflammation may lead to initiate angiogenesis and cancer progression [77]. Synthetic drug targeting is a monotargeted approach may not be functional due to the complexity of cancer; therefore, the best mode of action is to target several receptors to inhibit cell multiplication. There has been debate that multi-target therapies can mitigate the difficulties caused by acquired resistance to chemotherapeutic drugs [78]. The selected natural chemicals undergo docking to overcome these challenges and explore their anticancer potential for better treatment strategies [79]. Before conducting additional experiments, the computational approach assessed the binding affinities of possible drug candidate against target proteins that are associated with disease progression [80]. Phytocompounds may target various deregulated signaling pathways, including NF-кB, PI3K/mTOR, MAPKs, AMPKs, TGF-β, Wnt/β-catenin and JNK/STAT3 pathways in various cancers [81]. As we know, the HCC tumor microenvironment is a complex and dynamic system of stromal and immune cells communicating with tumor cells, and novel immunotherapeutics are required to target and manage resistance observed during treatment [82]. Current evidence suggests that TNF-α is an inflammatory mediator with a molecular connection between sustained inflammation and the emergence of several malignancies [83].

Additionally, research demonstrated that elevated TNF-α expression levels were observed in HCC liver tissues, and anti-TNFα treatment may suppress the progression of HCC [84, 85]. TGF-β is significantly enhanced in several malignancies, including HCC. A recent study revealed that inhibition of TGF-β increases the cytotoxic efficacy of therapies by targeting the EGFR [86] while TGF-βR1 is a key receptor for its activation. Another research reported that ginseng extract has an anti-fibrotic impact by downregulating the TGF-βR1 and TGF-β/Smad pathway [87]. Moreover, nitric oxide is excessively expressed in neoplastic lesions, is generated by iNOS during inflammation, and is involved in cancer progression [88]. It has been reported that iNOS is overexpressed in HCC and patients with fibrosis, cirrhosis, and hepatitis [89]. Dealing with these inflammatory mediators and their underlying pathways in preclinical studies to inhibit hepatocarcinogenesis, may suggest its therapeutic role in treating inflammation-induced hepatocellular carcinoma. So, we selected the target proteins such as TNF-α, TGF-β and iNOS having significant roles in HCC development. In present-day research, the binding energies of selected bioactive phytocompounds against selected target macromolecules are shown in Table 7. Overall, among selected drug-like compounds of both extracts of *B. monnieri*, the Spiro[(tricyclo[6.2.2.0(2,7)]dodeca-5,9- diene)-4,1’-cyclobutane]-11,2’-dione, 1,3,3,5,12,12-hexamethyl from BME extract exhibited the best-hit score against all target proteins, followed by caryophyllene oxide selected from BMH extract. The present study showed a valuable in-sight into the anticancer potential of BME extract, but further research is needed to explore their effective therapeutics before integrating them into the final product. A selected phytocompound with improved stability, bioavailability, and cell-directed delivery should be investigated in animal models. Subsequently, these compounds undergo clinical trials to verify their toxicity, safety and efficacy profile prior to regulatory authorization. The best-hit compounds of BME extract with significant antiproliferative potential, are selected due to their strong potency in the development of alternative anticancer drugs.

## Conclusion

In our study, *in vitro* and *in silico* approaches were used to evaluate the hepatoprotective potential of ethanol and n-hexane extracts of *B. monnieri*. BME extract was found to be more active in suppressing proliferation and inhibiting apoptosis by reducing oxidative stress due to the presence of valuable phytochemicals. Furthermore, the *in silico* attempt further confirmed the multi-targeted anticancer effect of phytochemicals identified in BME extract through the GCMS analysis. However, further investigations are needed at molecular and metabolic levels to explore its therapeutics against liver cancer before the development of novel plant-based drugs.

## Abbreviations

GCMS: Gas chromatography/Mass spectrometry
TNF-α: Tumor necrosis factor-alpha
TGF-β: Transforming growth factor-beta
TGF- β R1: Transforming growth factor-beta receptor 1
SD: Standard deviation
MTT: (3-[4,5-dimethylthiazol-2-yl]-2,5-diphenyl tetrazolium bvromide
DPPH: 2,2-diphenyl-1-picrylhydrazyl
